# Effect of tracer arrival time on the estimation of the myocardial perfusion in DCE-CMR

**DOI:** 10.1186/1532-429X-14-S1-P16

**Published:** 2012-02-01

**Authors:** Niloufar Zarinabad, Gilion Hautvast, Marcel Breeuwer, Eike Nagel, Amedeo Chiribiri

**Affiliations:** 1 Division of Imaging Sciences and Biomedical Engineering, King’s College London, London, UK; 2 Imaging Systems - MR, Philips Healthcare, Best, Netherlands; 3 Biomedical Engineering, Biomedical Image Analysis, Eindhoven University of Technology, Eindhoven, Netherlands

## Background

An accurate segmental myocardial blood flow (MBF) quantification can be performed by means of signal deconvolution techniques. The accuracy of MBF estimates relies on the precise identification of the tracer arrival time in the myocardium (tOnset). Voxelwise MBF quantification is likely to be more subject to such error than segmental MBF analysis due to the lower signal-to-noise ratio of myocardial signal intensity curves. Automated tOnset detection methods would be therefore warranted. The aim of this study was to assess the importance to of tOnset identification on voxelwise MBF quantification and to describe an automated algorithm to detect the optimal tOnset which minimizes the error in MBF estimates.

## Methods

Perfusion data were obtained from an hardware perfusion phantom (validated MBF 5 ml/g/min) and from patients during adenosine-induced hyperaemia (140µg/kg/min) using 0.075mmol/kg Gadobutrol (Gadovist, Schering, Germany) injected at 4ml/minute followed by a 20 ml saline flush. A pre-bolus technique was used for quantification.

Images were acquired on a Philips Achieva 3T (TX) system, equipped with a 32-channel cardiac phased array receiver coil (Philips, Best, Netherlands) with a saturation recovery gradient echo method (TR/TE 3.0ms/1.0ms, flip-angle 15°; effective k-t SENSE acceleration 3.8, spatial resolution 1.2x1.2x10mm, saturation-recovery delay 120 ms).

Data were analysed using a made-in-house software, which uses second derivative test followed by an iterative Fermi deconvolution which repeats the deconvolution for values of tOnset±4 heart beats from the tOnset value obtained from derivative test to identify the optimal tOnset that minimize the error between the actual myocardial curve and the fitted curve obtained after deconvolution. The results of perfusion estimation with a visually selected tOnset and with an optimized tOnset were compared.

## Results

**Figure 1:**
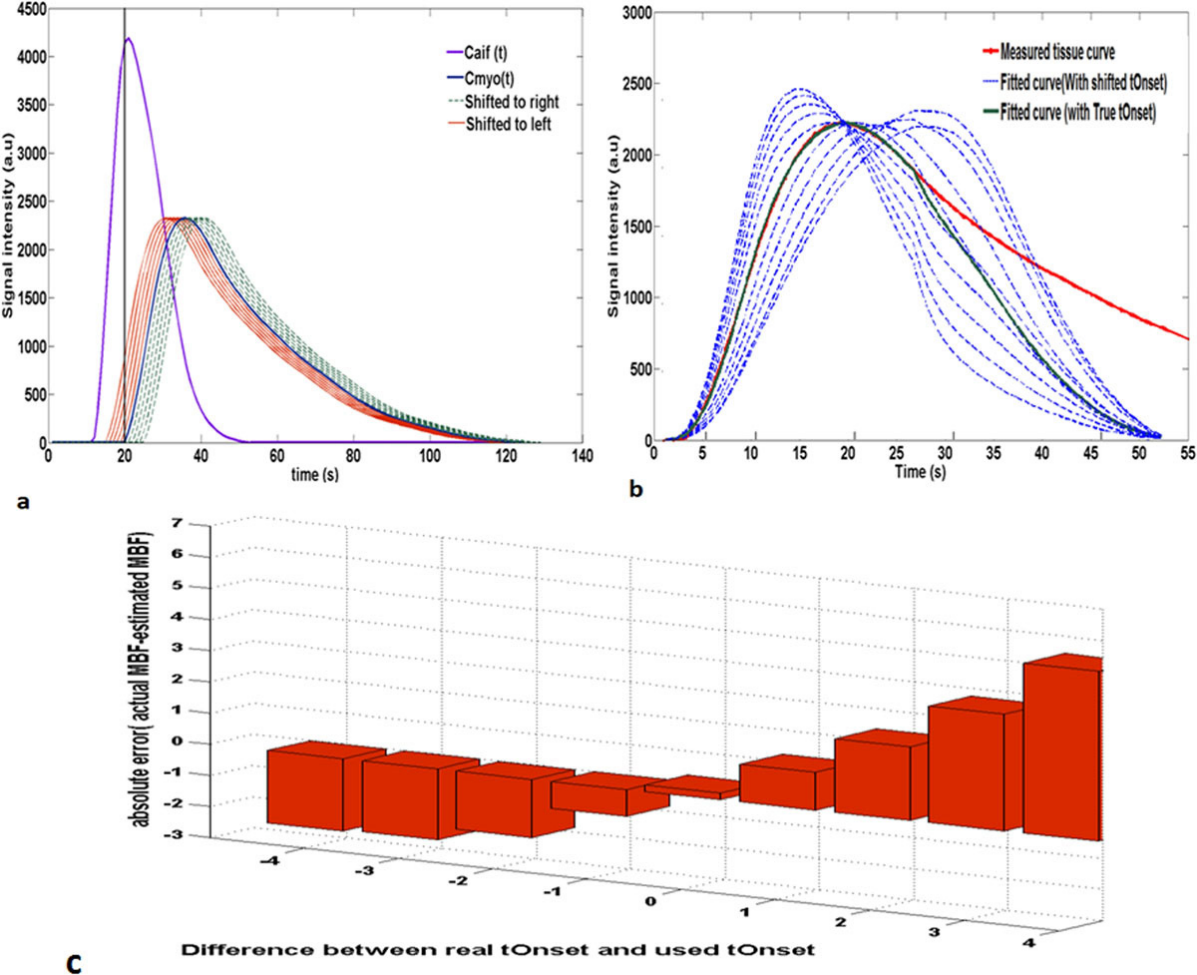
(a) Arterial input (Caif(t)) and myocardial curves (Cmyo(t)) in perfusion phantom. Cmyo(t) has been shifted artificially to right and left. (b) The fitted Cmyo(t) along with the actual one. The best fit has been obtained when the real tOnset is used. (c) The absolute error in estimation of flow vs. time shifts of tOnset.

Figure 1 shows the MBF absolute error (actual flow - estimated flow) vs. timing shifts plotted for the perfusion phantom. The lowest absolute error and the best curve fit have been obtained when the optimized tOnset is used.

**Figure 2:**
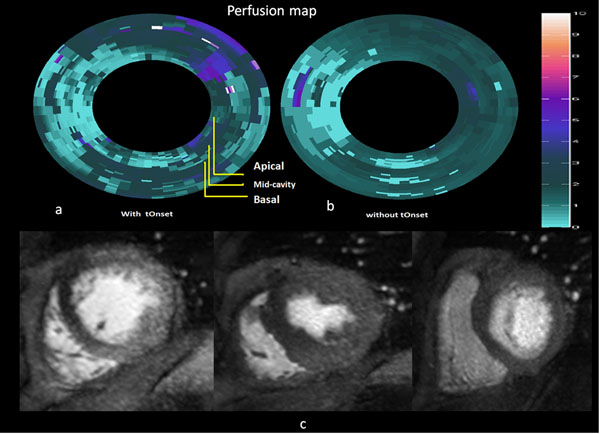
Perfusion maps obtained with and without using tOnset in a patient with chronic total occlusion of the LAD collateralized by the LCX. The LCX itself presents a 75% stenosis (FFR 0.65). Ischemia in its territory was not identified when a fixed tOnset was used for quantitative analysis.

Figure 2 shows the voxels wise perfusion map in a patient with occluded left anterior descending coronary artery and a significant coronary artery lesion on the left circumflex artery (fractional flow reserve 0.65) and symptoms of angina. tOnset estimation has been used for the analysis of perfusion in the left map which resulted in the identification of perfusion abnormalities in both the left anterior descending and left circumflex artery perfusion territories. The latter was missed in an analysis with fixed tOnset (Fig. 2.b).

## Conclusions

Perfusion estimates based on DCE-CMR have many desirable characteristics including high sensitivity in identifying the tissue at risk, however MBF estimates are biased by many factors including the tracer arrival time into myocardium tissue, and therefore attempts to quantify MBF without using the tOnset may be premature.

## Funding

The authors acknowledge financial support from the Department of Health via the National Institute for Health Research (NIHR) comprehensive Biomedical Research Centre award to Guy's and St Thomas' NHS Foundation Trust in partnership with King's College London.

